# The characteristics of eating, drinking and oro-pharyngeal swallowing difficulties associated with repaired oesophageal atresia/tracheo-oesophageal fistula: a systematic review and meta-proportional analysis

**DOI:** 10.1186/s13023-024-03259-x

**Published:** 2024-07-04

**Authors:** Alexandra Stewart, Roganie Govender, Simon Eaton, Christina H. Smith, Paolo De Coppi, Jo Wray

**Affiliations:** 1 Department of Language and Cognition, University College London, Chandler House,2 Wakefield Street, London, WC1N 1PF UK; 2 Great Ormond Street Hospital for Children, Great Ormond Street, London, WC1N 3JH UK; 3 Head and Neck Academic Centre, Division of Surgery and Interventional Science, University College London, Charles Bell House, 43-47 Foley Street, London, W1W 7TS UK; 4 University College London Hospital, 250 Euston Road, London, NW1 2PG UK; 5 Stem Cells and Regenerative Medicine Section, University College London Institute of Child Health, 30 Guilford Street, London, WC1N 1EH UK

## Abstract

**Introduction:**

Eating, drinking and swallowing difficulties are commonly reported morbidities for individuals born with OA/TOF. This study aimed to determine the nature and prevalence of eating, drinking and oro-pharyngeal swallowing difficulties reported in this population.

**Method:**

A systematic review and meta-proportional analysis were conducted (PROSPERO: CRD42020207263). MEDLINE, EMBASE, CINAHL, Pubmed, Scopus, Web of Science databases and grey literature were searched. Quantitative and qualitative data were extracted relating to swallow impairment, use of mealtime adaptations and eating and drinking-related quality of life. Quantitative data were summarised using narrative and meta-proportional analysis methods. Qualitative data were synthesised using a meta-aggregation approach. Where quantitative and qualitative data described the same phenomenon, a convergent segregated approach was used to synthesise data.

**Results:**

Sixty-five studies were included. Six oro-pharyngeal swallow characteristics were identified, and pooled prevalence calculated: aspiration (24%), laryngeal penetration (6%), oral stage dysfunction (11%), pharyngeal residue (13%), nasal regurgitation (7%), delayed swallow initiation (31%). Four patient-reported eating/drinking difficulties were identified, and pooled prevalence calculated: difficulty swallowing solids (45%), difficulty swallowing liquids (6%), odynophagia (30%), coughing when eating (38%). Three patient-reported mealtime adaptations were identified, and pooled prevalence calculated: need for water when eating (49%), eating slowly (37%), modifying textures (28%). Mixed methods synthesis of psychosocial impacts identified 34% of parents experienced mealtime anxiety and 25% report challenging mealtime behaviours reflected in five qualitative themes: fear and trauma associated with eating and drinking, isolation and a lack of support, being aware and grateful, support to cope and loss.

**Conclusions:**

Eating and drinking difficulties are common in adults and children with repaired OA/TOF. Oro-pharyngeal swallowing difficulties may be more prevalent than previously reported. Eating, drinking and swallowing difficulties can impact on psychological well-being and quality of life, for the individual and parents/family members. Long-term, multi-disciplinary follow-up is warranted.

**Supplementary Information:**

The online version contains supplementary material available at 10.1186/s13023-024-03259-x.

## Introduction

Oesophageal atresia (OA) with or without trachea-oesophageal fistula (TOF) is a rare, congenital abnormality caused by incomplete separation of the foregut in early embryonic development [1]. Following surgical repair, survival rates for infants born in developed nations are over 90% [1]. Life-long respiratory and gastroenterological morbidities are well-documented [1, 2].

Difficulty swallowing, or dysphagia, is a well-known complication of OA. Recent reviews have identified swallowing difficulties arising from oesophageal dysmotility, anastomotic stricture, eosinophilic oesophagitis, anastomotic leak, recurrent tracheo-oesophageal fistula and oro-pharyngeal dysphagia [3, 4]. This dysfunction correlates well with the fact that swallowing impairment is evident even during fetal life, possibly affecting function beyond the oesophagus [1]. Comella and colleagues undertook a systematic review of oesophageal morbidity, reporting median prevalence rates for a range of outcomes, and highlighting frequent presentation of oesophageal dysmotility (76%), oesophagitis (46%) and anastomotic stricture (29%). They reported a median dysphagia prevalence of 43% [4].

Dysphagia does not always arise from oesophageal morbidity. It can result from impairment of oral, pharyngeal, laryngeal, and respiratory anatomy and/or physiology [5]. This type of dysphagia has been less widely reported in those born with OA but has been suggested as an under-identified cause of respiratory morbidity in this population [3]. Comella’s systematic review of oesophageal morbidity did not aim to differentiate between oro-pharyngeal dysphagia and oesophageal dysphagia [4].

Swallowing difficulties have the potential to negatively impact on respiratory status, because of aspiration, but also impact on an individual’s experience of eating, drinking and mealtimes. This, in turn, can impact on quality of life [5]. The disruption to eating, drinking and mealtimes impacts not only the individual with dysphagia, but also parents, caregivers and other family members [6].

Assessment of dysphagia can, therefore, focus on the impairment, oro-pharyngeal and/or oesophageal, or can assess eating and drinking more broadly, including the psychosocial impacts, and the adaptations made to mealtimes to mitigate for swallow impairment. Each are of value but must be recognised for what they are. Self-perceived dysphagia differs from biomechanical dysphagia. Assessments of dysphagia can be “instrumental” i.e. those that use an instrument to assess swallowing, such as radiological imaging or endoscopy, or “non-instrumental” i.e. those that use patient or parent report to assess swallowing, such as a questionnaire or interview. Instrumental assessment describes impairment, whereas non-instrumental assessment describes perceived impairment, or the results thereof.

Comella and colleagues systematically reviewed the prevalence of oesophageal morbidities associated with OA. They also reported broad prevalence of dysphagia [4]. This current systematic review aimed to expand understanding of the nature of dysphagia and eating and drinking difficulties in those born with OA describing:

- The prevalence and characteristics of oro-pharyngeal swallow impairment in OA/TOF as identified by instrumental assessment
- The prevalence and characteristics of patient/carer reported eating and drinking difficulties in OA/TOF
- The psychosocial impact of altered eating and drinking on an individual born with OA/TOF and their caregivers.

## Methods

The study protocol was registered on PROSPERO (no. CRD42020207263.) and PRISMA guidelines followed.

### Search strategy

The following search strategy was applied to MEDLINE: -(Oesophageal Atresia/ OR Tracheoesophageal Fistula/ OR tracheoesophageal fistula OR tracheooesophgeal fistula OR trachea-esophageal fistula OR tracheo-oesophageal fistula OR oesophageal atresia OR esophageal atresia) AND (Deglutition/ OR exp Deglutition Disorders/ OR “Feeding and Eating Disorders of Childhood”/ OR Feeding Behaviour/ OR deglutition OR dysphagia OR feed* OR swallow*). Appropriate syntax alterations were made for searches in EMBASE, CINAHL, Pubmed, Scopus and Web of Science databases. The following databases were also searched: International Standardised Randomised Controlled Trials Number, clinicaltrials.gov. and Open Access Theses and Dissertations. Reference lists were hand searched.

Inclusion/exclusion criteria are presented in Table 1.

**Table 1:** Inclusion/exclusion criteria

Inclusion criteria	Exclusion criteria
Empirical study of feeding or oro-pharyngeal swallowing function using instrumental or non-instrumental (questionnaire) assessment, and/or, qualitative evaluation of feeding or swallowing outcome	Studies of oesophageal phase of swallowing (e.g. oesophageal manometry, pH impedance, gastric emptying, oesophageal/gastric endoscopy)
Includes participants with repaired congenital oesophageal atresia and/or trache-oeosphageal fistula	Studies where OA/TOF is not reported separately i.e. cannot be distinguished from other conditions
Year of publication-1990–2020	Studies in which feeding/swallowing outcome has not been evaluated using an instrumental or validated non-instrumental tool or qualitative methods e.g., feeding outcome described as oral/non-oral only
Written in English language	Studies relating only to acquired tracheo-oesophageal fistula, such as button battery ingestion
Published in peer reviewed journal or grey literature (e.g. theses). Including: Ahead of Print, In-Process; Other Non-Indexed Citations	Review, opinion or commentary only
	Conference proceedings

### Study selection

Searches were undertaken in January 2023 by AS and uploaded to Covidence (Covidence systematic review software, Veritas Health Innovation, Melbourne, Australia. Available at www.covidence.org.) for duplicate removal, screening and data extraction. Each article was screened (title/abstract) by two members of the team (AS plus either RG, JW, CS, SE or PDC). All studies rated “include” by at least one reviewer were included for full text screening, which was also conducted by two reviewers as above. Conflicts were resolved through consensus discussion at full text screening.

### Data extraction

Data extraction was conducted by AS with 20% of all studies checked by another member of the team. Further checking by a second reviewer was not deemed necessary as no systematic errors were identified.

All studies were reviewed for any data relating to a characteristic of oro-pharyngeal swallow function or eating/drinking experience. Details of all the data extracted are provided in Supplementary material 1. Data were exported to Excel (Microsoft® Excel® for Microsoft 365 MSO (Version 2208 Build 16.0.15601.20818)) for analysis.

### Definitions

Definitions for swallow and mealtimes characteristics used in this study are provided in Table 2.

**Table 2:** Swallow and mealtime characteristic definitions

Aspiration	Food or drink entering the trachea during swallowing
Laryngeal penetration	Food or drink entering the laryngeal vestibule but remaining above or at the level of the vocal cords
Oral stage dysfunction	Difficulty with preparing or transporting the bolus in the mouth
Pharyngeal residue	Food or drink remaining in the pharynx after swallowing
Nasal regurgitation	Food or drink entering the naso-pharynx or nasal cavity during swallowing
Delayed swallow initiation	Bolus dwelling in the pharynx prior to swallow initiation
Difficulty swallowing solids	Any reported difficulty swallowing any type of food
Difficulty swallowing liquids	Any reported difficulty swallowing liquids
Odynophagia	Pain on swallowing
Coughing/choking when eating	Reported coughing or choking *during* eating or drinking
Need for water	Any report of needing sips of fluid to aid bolus clearance when eating
Prolonged mealtimes	Any report of slow eating or feeding, mealtimes lasting over 30 min, slower to eat than peers
Need for texture modification	Any report of avoiding certain food textures or altering food or drink texture to aid swallowing
Challenging mealtime behaviour	Parent report of excessive food refusal or selectivity, the need for distraction, difficulty sitting at a table or excessive passivity at mealtimes
Avoiding eating with friends	Any action taken to avoid social aspects of eating or drinking
Increased parent anxiety	Any report of parent anxiety, worry or stress specifically at mealtimes

### Quality assessment

The Mixed Methods Appraisal tool (MMAT) [7] was used to assess the quality and risk of bias of each paper. The appropriate MMAT tool for study type was adopted. Percentage of elements achieving a “yes” was used to assess overall study quality.

### Data synthesis

#### Quantitative

Prevalence ranges for each oro-pharyngeal swallow and eating/drinking/mealtime characteristic were calculated. Results from observational studies were included in a binary random effects DerSimonian-Laird meta-proportional analysis (proportion and 95% confidence interval) using Open Meta Analyst software [8]. To reduce the risk of selection bias, intervention studies were excluded from the prevalence meta-analysis.

Due to the inconsistent reporting of age and OA subtype, meta-analysis of these subgroup was not possible. Therefore, narrative synthesis was conducted to explore the impact of age and OA subtype/repair type on swallow/eating/drinking characteristics.

#### Qualitative

As per Joanna Briggs institute guidelines, qualitative data was synthesised using a meta-aggregation approach [9]. Author interpretations (“findings”) were aggregated into “categories” and an explanatory statement generated. Only findings that could be substantiated with data were deemed credible and included. Unsupported evidence was not included in the meta-aggregation.

#### Mixed methods synthesis

Where quantitative and qualitative data describe the same phenomenon, a convergent segregated approach was used to synthesise data meaning that different types of data were synthesised separately, then integrated [10]. This enabled greater depth of understanding from which recommendations for practice were generated.

## Results

**Fig. 1:**
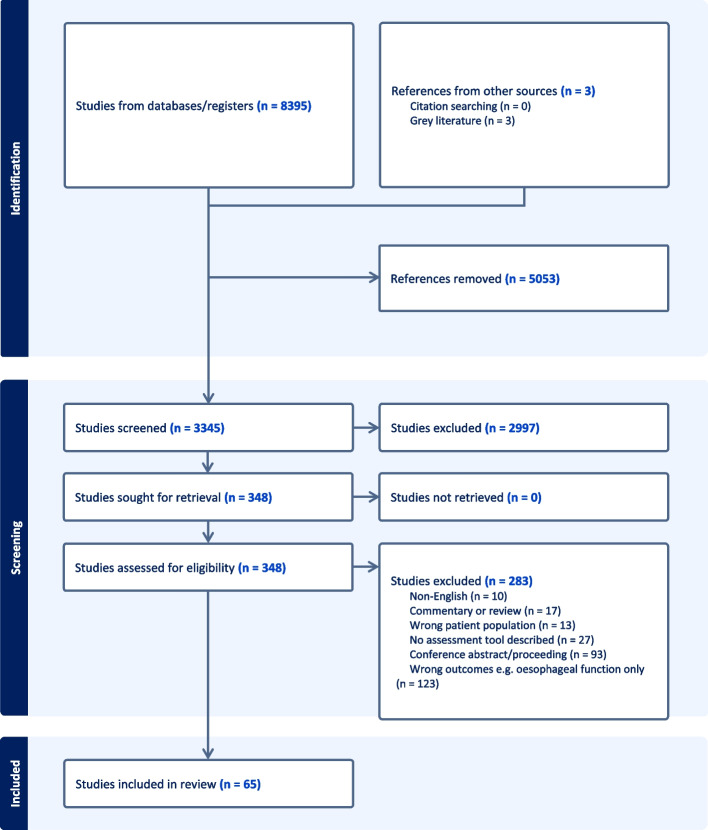
PRISMA flowchart outlining study selection

A total of 65 studies were included in this review. The selection process is summarised in Fig. 1. A summary table of study types, populations and quality assessment is provided in Supplementary material 2 for all included studies. In general, the quality of the studies included was low. Most data were extracted from observational studies: case series (*n* = 20), cross-sectional studies (*n* = 37) and case report (*n* = 1). There was only one randomized control trial, one non-randomized trial, and one cohort study. There were two case control studies and three qualitative studies. Most studies were conducted in Europe (*n* = 43), followed by North America (*n* = 14), Australia (*n* = 5) and Asia (*n* = 2). One study was conducted in several countries. Thirty-six studies had fewer than 50 participants, 12 studies had 50–100 participants and 17 studies had over 100 participants. Forty-four studies reported repair type: all repair types (*n* = 20), primary repair only (immediate or delayed) *(n* = 22), oesophageal replacement only (*n* = 2). Studies included participants of different age ranges: < 4 years (*n* = 11), 0–18 years (*n* = 27), > 18 years (*n* = 11). Fifteen studies included both children and adults of any age. One study did not report participant age.

Fifteen studies met over 80% of the MMAT criteria for their respective study design, indicating higher quality with lower risk of bias. Nineteen studies met less than 50% of the MMAT criteria, indicating lower quality or higher risk of bias. Analysis indicated selection bias as a frequent risk, typically as studies were conducted at specialist referral centres with higher than expected numbers of non-type C OA subtypes or non-consecutive case reporting.

### Oro-pharyngeal swallow impairment (instrumental assessment)

Fifteen studies used instrumental assessment (videofluoroscopy *n* = 12, fibreoptic evaluation of swallowing *n* = 1, videomanometry *n* = 1, oral pharyngeal motility study *n* = 1) to characterise oro-pharyngeal swallow function. One paper included assessment of adults born with OA/TOF [11]. Five out of 15 included only children under 4 years of age.

Prevalence ranges and pooled prevalence rates for each oro-pharyngeal swallow characteristic are provided in Table 3. One study presented swallow characteristics for a group of children undergoing intervention for pharyngeal dysphagia, these were not included in the pooled prevalence calculations [12].

**Table 3:** Prevalence range and pooled prevalence for oro-pharyngeal swallow characteristics from instrumental assessments

Characteristic (number of studies)	Total participants	Prevalence range	Pooled prevalence (95% CI)	Heterogeneity (I^2^)
Aspiration (*n* = 14)	896	0–1.00 all studies 0.8–0.45 case series only	0.24 (0.18, 0.31)	79% Substantial
Laryngeal penetration (*n* = 9)	599	0–0.13	0.06 (0.01, 0.11)	76% Substantial
Oral stage dysfunction (*n* = 6)	118	0–0.50	0.11 (0.02, 0.21)	66% Substantial
Pharyngeal residue (*n* = 7)	188	0.05–0.38	0.13 (0.04, 0.21)	74% Substantial
Nasal regurgitation (*n* = 4)	150	0.08–0.16	0.07 (0.00, 0.13)	63% Substantial
Delayed swallow initiation (*n* = 4)	118	0.16–0.75	0.31 (0.11, 0.50)	85% Substantial
No pharyngeal deficit on VFSS (*n* = 2)	177	0.39–0.72	0.55 (0.23, 0.87)	92% Substantial

Seven studies used categorical rating scales for various components of swallow physiology [12–18]. Six studies reported binary aspiration/no aspiration outcome only [11, 19–23]. One videofluoroscopy study used quantitative methods to measure hyolaryngeal elevation [24]. One study used low resolution manometry to quantitate upper oesophageal sphincter pressures and timing, pharyngeal constriction, and bolus transit time [11].

**Fig. 2:**
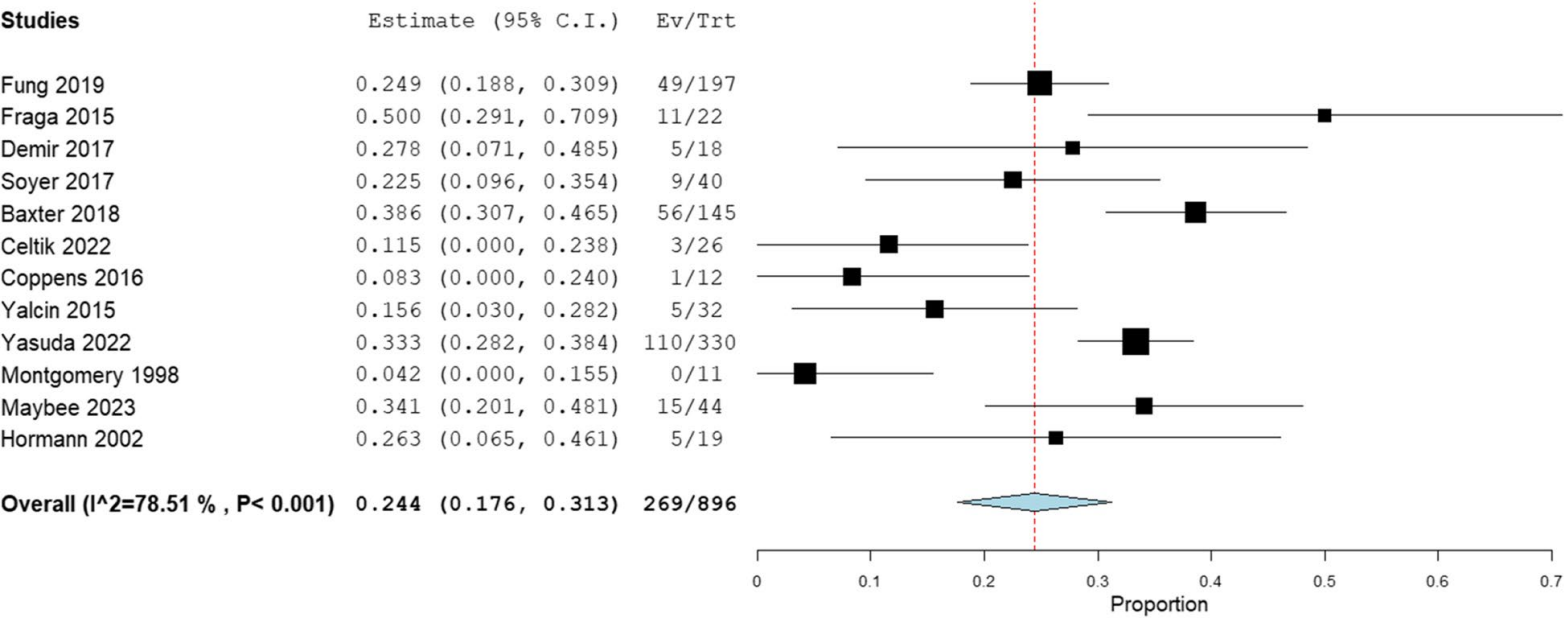
Forest plot for pooled prevalence of aspiration detected on instrumental assessment

The Penetration-Aspiration scale (PAS) [25] was used in five studies as a validated measure of aspiration/penetration [12, 13, 18, 24, 26]. Two studies reported median PAS scores [12, 13]. One was the intervention study in which the median PAS was 8 (material enters the trachea with no attempt to clear) for liquids and 1.5 (no entry of material into the larynx or trachea) for solid foods [12]. Soyer and colleagues used the PAS to report swallow characteristics in a single centre case series [13]. Median PAS scores were 1 for all children, other than liquid swallows for children with delayed primary repair, where the median PAS was 2 (entry of material into the larynx with clearing). Other reported PAS scores were converted into % of aspiration or penetration, included in Table 3. A forest plot details the pooled prevalence for aspiration in Fig. 2.

Two studies compared aspiration rates between repair or OA types. Celtik and colleagues reported that all children with aspiration in their cohort had long gap OA [15]. Soyer and colleagues reported more frequent aspiration of liquids for those with delayed primary repair, compared to those with oesophageal replacement and more frequent aspiration of “pudding” consistency for those with delayed primary repair, compared to those with early primary repair [13].

Seven studies reported rates of pharyngeal residue, four of which specified place of residue within the pharynx [12, 13, 16, 18]. Residue was reported in the valleculae, pyriform sinuses and on the pharyngeal wall i.e. throughout the pharynx. Celtik and colleagues reported significantly higher rates of residue in children born with long gap OA, compared to short gap OA [15]. Soyer and colleagues found no differences between those with primary repair and oesophageal replacement [13].

### Patient/parent-reported eating and drinking difficulties (non-instrumental assessment)

Patient or parent-reported eating and drinking difficulties have been divided into two categories: 1) swallowing difficulties, 2) adaptations to eating/drinking (i.e. implied swallowing difficulty).

#### Assessment tools

Forty-seven studies used non-instrumental methods. Twenty-three of these studies used validated assessment tools, twenty-four unvalidated, author-generated assessment tools.

#### Validated assessment tools

Thirteen different validated tools were used across all studies, summarised in Table 4.

**Table 4:** Summary of validated assessment tools

Authors	Assessment tool	Summary of tool characteristics
Harrington 2021 [27] Thompson 2021 [28] Coppens 2016 [16] Van Tuyllvan 2021 [29] Celtik 2022 [15] Bourg 2022 [30] Yasuda 2022 [23] Baxter 2018 [22] Soyer 2017 [26]	Functional oral intake scale	7-point, clinician-rated measure of oral and non-oral intake. Validated initially for adults with post-stroke dysphagia [15, 26, 27, 29] Author adapted versions [16, 23, 28] Paediatric adapted versions [22, 30]
Barni 2019 [31] Birketvedt 2020 [32] Capitanio 2021 [33] Mikklesen 2022 [34] Soyer 2017 [26]	EAT-10	10-item patient-reported screening tool, validated for adults with dysphagia. Components: swallowing impairment, swallowing-related QOL, weight gain [33, 34] Validated paediatric version (Pedi-EAT-10) [26, 31] Author adapted version for OA [32]
Gatzinsky 2011 [35] Yalcin 2015 [18] Traini 2022 [36] Soyer 2017 [26]	Dakkak dysphagia score	Patient- or parent-rating of ability to swallow nine food/drink textures. Validated for use with adults
Baird 2015 [37] Menzies 2020 [38] Pham 2022 [39] Traini 2022 [36]	Montreal children’s hospital feeding scale	18-item, parent-rated tool. Components: oro-motor function, mealtime length, mealtime behaviour and psychosocial impact. Validated in children 6 months-6 years
Dellenmark-Blom 2020 [40] Dellenmark-Blom 2022 [41]	EA-QOL questionnaire	18-item, parent-rated tool (validated for children aged 2–7 years). Components: eating, physical health and treatment, social isolation and stress 26-item parent or child-rated tool (validated for children aged 8–17 years). Components: eating, social relationships, body perception and health, wellbeing
Serel Arslan 2018 [42]	Karaduman chewing performance scale	5-point clinician-rated scale of chewing function. Validated children aged 2–15 years
Serel Arslan 2018 [42]	International dysphagia diet standardisation initiative	7-point clinician-rated scale of food and drink texture descriptions. Validated for all ages
Tan 2015 [43]	Atkinson swallow scale	5-point scale of ability to eat food/drink textures. Not clear if clinician or patient reported. Reliability/validity not reported
Serel Arslan 2020 [44]	Turkish feeding-swallowing impact survey	18-item parent-rated assessment of feeding-related quality of life. Validated for children aged 1–12
Ax 2021 [45]	Oesophageal Atresia feeding survey	9-item parent-rated author developed assessment of strategies used to mitigate feeding difficulties. Used for children aged 2–17 years. Reliability/validity not reported
Dellenmark-Blom 2019 [46]	OA coping questionnaire	9-item parent- or child-rated assessment of mealtime coping strategies. Condition-specific. Validated for children aged 2–17 years
Bergmann 2022 [47]	pedsSWAL-QOL	32-item parent-rated assessment of swallowing-related quality of life. Adapted from adult tool. Reliability/validity not reported
Gibreel 2017 [48]	Swallow dysfunction questionnaire	29-item patient-rated assessment of dysphagia. Components: ability to manage 5 food/drink consistencies, swallowing “habits”, eating/drinking quality of life. Validated for adults with OA

### Patient-/parent-reported characteristics of swallow impairment

Five patient or parent-reported characteristics were related to swallow impairment: difficulty swallowing solids, difficulty swallowing liquids, oral stage dysfunction, odynophagia and coughing when eating. Prevalence ranges and pooled prevalence rates for each swallowing characteristic are presented in Table 5.

**Table 5:** Prevalence ranges and pooled prevalence for patient-/parent-reported swallowing characteristics

Characteristic (number of studies)	Total participants	Prevalence range	Pooled prevalence (95% CI)	Heterogeneity (I^2^)
Difficulty swallowing solids (*n* = 14)	599	0.33–0.70	0.45 (0.36, 0.54)	82% Substantial
Difficulty swallowing liquids (*n* = 8)	303	0.15–0.27	0.06 (0.02, 0.10)	60% Substantial
Oral stage dysfunction (*n* = 3)	167	0.10–1.0 all studies 0.10–0.35 case series only	n/a	
Odynophagia (*n* = 4)	111	0.13–0.73	0.30 (0.10, 0.50)	82% Substantial
Coughing or choking when eating (*n* = 6)	390	0.15–0.45	0.22 (0.13, 0.31)	78% Substantial
No deficit (*n* = 21)	2071	0.15–0.85	0.38 (0.28, 0.48)	95% Substantial

#### Difficulty swallowing solids

**Fig. 3:**
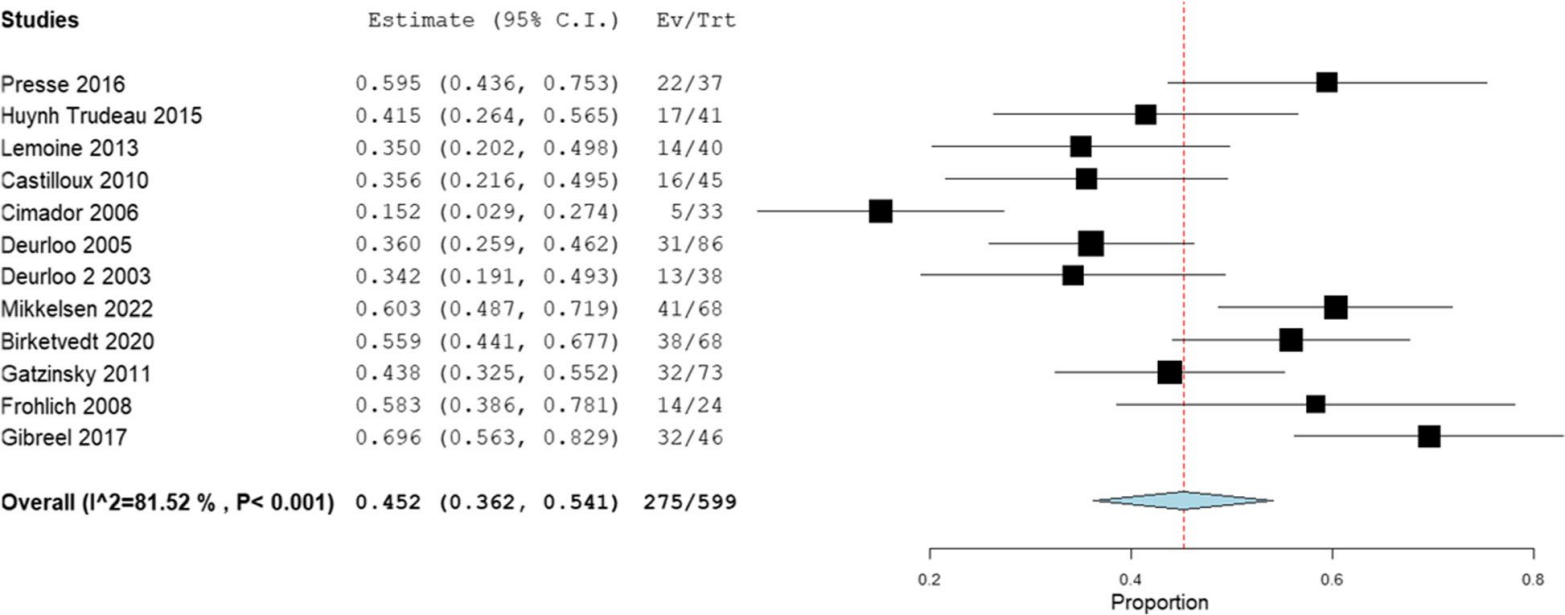
Forest plot for pooled prevalence of difficulty swallowing solids derived from non-instrumental assessment methods

Difficulty swallowing solids was the most reported swallow characteristic, described in 14 studies. Figure 3 is a forest plot detailing the pooled prevalence. The highest rate (70%) was from a study of adults, of whom 85% had type C OA [48]. Most studies included a wide range of ages and all OA types. No studies reported “long gap”, oesophageal replacement or non-type C OA prevalence separately. Maybee and colleagues [17] reported by age, identifying increasing prevalence of difficulty swallowing solids throughout the first four years of life.

Five studies included only adult participants. Pooled prevalence rate for these studies was 0.50 (0.37, 0.62). Eight studies included only children. Pooled prevalence rate for these studies was 0.43 (0.34, 0.52).

Four studies used Dakkak dysphagia score to assess difficulty with various food textures [18, 26, 35, 36]. Median scores ranged from 4.5–12. Gatzinsky and colleagues reported increasing frequency of difficulty with increasing food texture ie. yogurt least reported, meat the most frequently reported as difficult to swallow. Two studies compared Dakkak scores between OA subtypes [26, 35]. Both found significantly higher scores (more difficulty) in those with type A OA compared to type C OA. Soyer and colleagues reported higher scores for those with aspiration identified on VFSS compared to those without aspiration and those undergoing delayed OA repair compared to those with immediate repair [26].

Thirteen studies reported difficulty with specific food types. Meat was reported as difficult in 9/13 studies. Bread, rice and vegetables were also frequently reported as difficult to swallow.

#### Difficulty swallowing liquids

Eight studies reported prevalence of difficulty swallowing liquids [17, 29, 34, 35, 48–51]. Prevalence rates ranged from 1.5–27% (Table 5). In general, rates of swallowing liquids were lower than difficulty with solid foods. Gibreel and colleagues reported that 22% of adults had difficulty swallowing liquids but the frequency of difficulty was “rare” or “sometimes”. The highest prevalence of difficulty with liquids was reported by Maybee in children aged 0–6 months (27%), which reduced to 11% for those over 48 months. No studies reported difficulty by OA subtype or repair type.

#### Oral stage difficulties

Three studies reported the prevalence of oral stage difficulties using clinical observation [17, 42, 52]. One was an intervention study in which all children were identified as having oral stage (chewing) difficulties [52]. Two retrospective case series reported prevalence rates of 10–35% [17, 42]. Maybee and colleagues identified peak prevalence of oral stage difficulties at 24–48 months of age (35%) [17]. Serel Arslan and colleagues identified a significant correlation between time to starting oral feeding and chewing dysfunction, with mean time to starting oral feeding of 1 week for children without chewing disorder and 24 weeks for those with chewing disorder. There was no significant association between chewing function and repair type [42].

#### Odynophagia

Four studies reported odynophagia, with prevalence ranging between 13%-73% (Table 5). The study with the lowest reported rates included all ages [51], the highest included only adults [11]. Pain was reported by 25% of adults who had undergone oesophageal replacement [53].

#### Coughing/choking

Six studies reported coughing or choking on eating/drinking, with prevalence ranging between 15–45% (Table 5). The lowest rates were reported in a study of adolescents and adults [54], the highest in preschool-aged children [55]. Puntis et al. differentiated between rates of coughing on food vs drink and by repair type [56]. Coughing was most frequent on milk for children undergoing primary repair (32%). However, children requiring oesophagostomy coughed more frequently on solids (28%), than those with primary repair (16%).

#### No deficit

Twenty-one studies reported no eating, drinking or swallowing difficulties using non-instrumental tools. Four studies reported results for those with long-gap OA [27–30]. There was no difference in prevalence comparing those reporting only long-gap OA (38% (95% CI 22–55%)) with those reporting mixed OA subtypes (38% (95% CI 28–48%)). Lowest rates of “no deficit” (15%) were reported in a study of adolescents and adults, 85% of whom had type C OA [34]. Highest rates (85%) were reported in a study of children under 11 years of age, 95% of whom had type C OA [38].

### Patient-/parent-reported characteristics of eating and drinking adaptations

The most frequently reported alterations to eating and drinking were: a need to drink water to clear food, eating slowly and food texture modification. Prevalence ranges and pooled prevalence rates are presented for these characteristics in Table 6.

**Table 6:** Prevalence range and pooled prevalence for patient-/parent-reported eating and drinking adaptations

Characteristic (number of studies)	Total participants	Prevalence range	Pooled prevalence (95% CI)	Heterogeneity (I^2^)
Need for water when eating (*n* = 14)	930	0.31–0.75	0.49 (0.42, 0.57)	83% Substantial
Prolonged mealtimes/eating slowly (*n* = 15)	1015	0.05–0.88	0.37 (0.28, 0.46)	91% Substantial
Need for texture modification (*n* = 12)	753	0.05–0.54	0.28 (0.18, 0.38)	93% Substantial

#### Need for water to clear

Fourteen studies reported prevalence of using water to help clear food [32, 34, 45, 48–50, 53, 54, 57–62], with rates ranging from 31%[48]-75%[27]. Both the lowest and highest rates were reported in studies of adults. One study included only patients with long gap OA undergoing oesophageal replacement, reporting 5/8 participants (63%) using water to clear food [53]. No other studies reported OA subtypes of repair type separately.

#### Prolonged mealtimes

Fifteen studies reported the need to eat slowly or having prolonged feeds/mealtimes [17, 32, 34, 38, 39, 45, 49, 50, 53, 56, 57, 61–64]. Prevalence ranged from 5–88%. The lowest prevalence was reported in children 0–6 months [17], the highest by adults who had undergone oesophageal replacement [53]. Puntis and colleagues reported those requiring oesophageal replacement separately from those undergoing primary anastomosis, identifying that mealtimes were less frequently prolonged in those undergoing oesophageal replacement than in those with primary repair (food only) [56].

#### Texture modification

Twelve studies reported modification of food textures as a strategy to mitigate for swallowing difficulty [15–17, 32, 45, 48, 50, 55, 59, 62, 63, 65]. Prevalence ranged from 5%[17]-54%[53]. The lowest rates were reported in children aged 0–6 months. Two studies included only adult participants, in which prevalence rates were 33% [65] and 30% [48]. No studies reported OA subtypes or repair type separately.

### Psychosocial aspects of eating and drinking

The psychosocial impact of eating and drinking difficulties for those born with OA/TOF and their parents/carers were described in studies using quantitative and qualitative methodologies, which are described separately and then synthesised.

#### Quantitative synthesis

##### Eating and drinking-related quality of life (those born with OA)

Although a number of quantitative studies used tools which included items related to eating and drinking quality of life (QOL), only three papers reported specific results [34, 62, 66]. Mikkelsen and colleagues reported 9% of adults born with OA/TOF avoided eating with friends as a result of their swallowing difficulties (Table 7). Dellenmark Blom (2019) noted that children with difficulty swallowing food more often avoided or expressed fear or worry about eating than those without difficulty [66]. Dellenmark Blom et al. (2022) reported scores from a disease-specific QOL tool, identifying no statistically significant differences in eating subscale scores between those with long-gap (delayed repair) and short-gap OA (immediate repair) [62].

**Table 7:** Prevalence rates and pooled prevalence for patient-/parent-reported psychosocial impact of eating and drinking difficulties

Characteristic (number of studies)	Total Participants	Prevalence range	Pooled prevalence (95% CI)	Heterogeneity (I^2^)
Avoiding eating with friends (*n* = 1)	68	0.09	n/a	n/a
Increased parent anxiety at mealtimes (*n* = 3)	216	0.39–0.57	0.34 (0.13, 0.56)	88% Substantial
Challenging mealtime behaviour (*n* = 4)	221	0.15–0.29	0.25 (0.19, 0.31)	0% Low

##### Eating and drinking-related quality of life (family members)

Six studies reported some aspect of parental stress or anxiety at mealtimes using quantitative measures [38, 39, 47, 55, 63, 67]. Frequent episodes of choking were associated with higher parental anxiety [55]. Fear of choking was higher in parents of children under 5 years than over 5 years [47]. Fear of choking resulted in parents not offering developmentally- or age-appropriate foods in 12/56 children [63]. Anxiety at feed times was found to negatively impact parent–child interaction [67]. Three studies reported prevalence rates for increased parent anxiety at mealtimes, generating a pooled prevalence rate of 34% (Table 7).

Four studies reported the impact of feeding or swallowing difficulties on family/parent quality of life more broadly using quantitative measures [31, 44, 47, 62]. Dellenmark-Blom reported a higher number of feeding difficulties correlated with lower scores on the PedsQL family impact module [41]. Using the Feeding-Swallowing Impact Survey (FSIS), Serel Arslan and colleagues identified significantly poorer feeding-related quality of life in those with isolated OA compared with OA/TOF and those with delayed repair compared to early repair [44]. There were moderate-strong correlations between FSIS scores and time to start oral feeding. Bergmann et al. reported severe impact on swallowing-related quality of life in only 3/44 (7%) children born with OA with very or extremely low birth weight [47]. Swallow-related quality of life was independent of OA type or surgery type (single vs staged repair).

##### Challenging mealtime behaviour

Four studies reported prevalence of extreme food selectivity or challenging mealtime behaviour (food refusal, distress) [17, 38, 39, 63]. Prevalence range and pooled prevalence are detailed in Table 7. Although three out of four studies included children of all ages, the mean age of participants was < 4 years in all studies.

##### Coping

Dellenmark-Blom [66] and colleagues quantitatively investigated use of coping strategies during eating and drinking in children born with OA [46]. They identified nine different coping strategies, such as recognising responsibility (“ I have learned what to do and can manage problems myself”) and acceptance (“I am used to my situation and have adjusted to what I can eat”), used by 77% of children. Children aged 2–7 used a mean of six strategies, children aged 8–18 used a mean of 5 (when self-reported), or 6 (when parent-reported). These were more commonly employed by children who experienced difficulties swallowing food.

#### Qualitative synthesis

Three qualitative studies examined psychosocial aspects of eating and drinking [68–70]. All recruited using patient support groups. Two studies investigated experiences of parents of children born with OA/TOF [68, 69]. One investigated experiences of adults who had been born with OA/TOF [70]. Five categories were developed from aggregated data and are summarised below with illustrative quotes.

##### Fear and trauma associated with eating and drinking

Those born with OA/TOF and those caring for them experience anxiety and fear of coughing/choking when eating and drinking. For some, a trauma response is triggered.

*“I constantly worry about eating if I don't have a drink nearby. Although I don't have as many symptoms as others might, it does cause me anxiety”* [70]

*“It was terrifying. […] I was so scared she would get stuck and choke.” *[68]

*“I think my first experience scarred me a little. Those earlier memories still haunt me and set me up to feel anxious about feeding…”* [69]

##### Isolation and a lack of support

Eating and drinking difficulties can cause those born with OA and those caring for them to avoid or have negative experiences in social situations. For parents a lack of support creates uncertainty about how to manage, increasing feelings of isolation.

“*I often avoid going out for meals or eating in crowded places due to worrying about how long it takes me to eat and having any issues in public. I also often feel as though friends and family may judge how slow I am at eating and I often become very anxious when eating in front of people.*” [70]

“*I felt completely on my own and isolated. Very little support or advice…very much on my own fighting to do the best I could every day*” [69]

*“The hard thing was the sole responsibility: no one else would ever feed her or look after her without me as the choking frightened them.”* [68]

##### Being aware and grateful

Parents of children born with OA/TOF acknowledged that getting through difficult times with eating and drinking difficulties had made them grateful for progress, no matter how small.

*“I never get tired of watching him eat. Little big steps….I’m surprised by what he can manage… I'm also surprised when he can't manage something that seems ok.”* [69]

*“I would not change him for anything that has happened as it has taught me to never take anything for granted.”* [68]

##### Support to cope

Parents acknowledged the support systems that enabled them to develop ways to cope with eating and drinking difficulties. This included friends and family, support groups and professionals.

*“I have a group of friends with babies around the same age and they are just wonderful while out and about.”* [69]

*“The Facebook TEF support group was a lifeline during this time! So many food suggestions and encouragement was given.”* [68]

*“I spent large parts of the day alone with baby and facing the fear of feeding […] without much support. Getting a SALT on board at this stage was probably more important for my mental welfare at this time than she realized.”* [68]

##### Loss

Parents experienced feelings of loss of normal feeding experiences as a result of their child’s eating and drinking difficulties.

“[Not being able to breastfeed her] *was hard for me because I felt that I had failed her. I did feel like her ‘baby-hood’ if you will, was stolen from her and I.”* [68]

#### Mixed methods synthesis

The available data from both quantitative and qualitative studies indicate that eating, drinking and swallowing difficulties have psychosocial impacts for those born with OA/TOF and those caring for them. Quantitative data indicates approximately 34% of parents experience mealtime anxiety. Qualitative data indicates that this arises from traumatic mealtime experiences, fear of choking, being unsure how to manage and feeling isolated. Quantitative and qualitative data indicate that coping and resilience for individuals and parents/carers develops through peer and professional support. While qualitative data highlights the occurrence of these difficulties, the limited quantitative evidence examining any psychosocial impact of eating, drinking or swallowing difficulties, particularly for adults born with OA/TOF, limits the ability to accurately determine their prevalence or the influence of medical/surgical factors on outcome (for example severity of swallow impairment or presence of gastro-oesophageal reflux).

## Discussion

This systematic review aimed to summarise and synthesise the current evidence for the prevalence and nature of swallowing, eating and drinking difficulties, and their psychosocial impact for those born with OA/TOF. The main findings of this review are discussed under key headings of oropharyngeal dysphagia and psychosocial impact in keeping with the above-mentioned aims.

### Oro-pharyngeal dysphagia

Pooled prevalence for aspiration caused by oro-pharyngeal dysphagia was 24%. This suggests that not all eating and drinking difficulties or aspiration-related respiratory disease in this population are caused by oesophageal dysfunction alone. Most studies used videofluoroscopy to evaluate swallow function, reporting aspiration/no aspiration as a binary outcome. A smaller number reported results of a categorical rating scale for various components of swallow function. None of the rating scales used demonstrated reliability or validity. While providing some evidence for the presence of oro-pharyngeal dysphagia the current evidence fails to demonstrate the underlying aetiology. Subjective rating scales provide potential biased assessment and rating observable consequences of swallow dysfunction fails to identify underlying cause. Why do these children aspirate?

Structural airway abnormalities are common in this population and become apparent after extensive evaluation [71]. In the studies examined here they were reported by three studies, reporting higher rates of oro-pharyngeal dysfunction in children with laryngeal cleft and vocal cord palsy [19, 20, 22]. While the former is usually congenital but can be diagnosed later in life, the latter is usually the consequence of recurrent laryngeal nerve injury at corrective surgery. Demir and colleagues suggested incomplete hyoid movement caused by a tethering effect may be an underlying cause of aspiration in this population [24]. These are all plausible explanations for aspiration. However, this review also demonstrated that approximately 30% of children with OA have delayed swallow initiation, 12% present with post-swallow residue and 6% experience nasal regurgitation. It is unclear whether these features of oro-pharyngeal dysphagia can be explained by a structural airway abnormality, or whether there could also be altered pharyngeal motility in addition to the high rates of oesophageal dysmotility.

The more recent use of high-resolution manometry in the field of deglutition has significantly improved understanding of oesophageal motility patterns [4]. This technology can also be used to assess pharyngeal function, providing quantitative assessment of velar and pharyngeal constriction, timing and efficiency of upper oesophageal sphincter opening and, when used with impedance, information regarding bolus flow [72]. One study used low resolution manometry in adults born with OA, identifying altered timing of bolus transit through the pharynx [11]. Ferris and colleagues used a cohort of children with OA/TOF without signs or symptoms of pharyngeal dysphagia to assess piecemeal deglutition in normal swallowing [73]. Use of this technology with a clinical cohort may improve understanding about the underlying aetiology of these oro-pharyngeal swallow patterns.

Other recent advances in the analysis of videofluoroscopy swallow studies may also help to accurately describe and improve understanding of oro-pharyngeal swallow dysfunction. Miles and colleagues describe methods for reliably obtaining quantitative measures of pharyngeal transit time, upper oesophageal sphincter opening, pharyngeal constriction, bolus clearance and coordination of airway closure [74]. Standardised, valid and reliable categorical rating scales are now widely used to assess adults with dysphagia but have not been used in the OA/TOF population to date [75]. Use of such methods in future studies would improve the quality of data available and our understanding thereof.

Current evidence is limited by a lack of natural history studies. Only one study using instrumental assessment measures involved adults born with OA/TOF, highlighting a significant gap in the literature [11]. Most data in this synthesis were generated by tertiary or referral centres, such as a research hospital or specialist aerodigestive clinic. Typically, investigation of oro-pharyngeal swallow function is initiated by symptom report or clinical observation prompting referral for videofluoroscopy. This selection bias is likely to have inflated reported prevalence of oro-pharyngeal swallowing difficulties.

### Patient-/parent-reported swallowing difficulties

Evidence from symptom questionnaire or patient report demonstrated a low prevalence of difficulty swallowing liquid (6%), compared to difficulty swallowing solid food (45%). This supports the notion that swallow dysfunction in OA is caused by oesophageal morbidity. However, this conflicts with evidence from this review which indicates up to 45% have some degree of oro-pharyngeal dysfunction and that, in this population, aspiration during the swallow is more likely to occur with liquids than food. Previous research suggests that in young children such questionnaires are poor at discriminating oesophageal from pharyngeal morbidity [76]. Mikkelsen and colleagues reported poor correlation between oesophageal metaplasia and symptom report in adolescents and adults with OA [34]. This review highlights the frequency with which adaptations to mealtimes and coping strategies are adopted by those with OA/TOF. Thus, an individual’s perception of swallow dysfunction, when so well adapted, may not be reflective of underlying physiology. While symptom report tools are valuable screening tools for swallowing/eating/drinking difficulties, instrumental assessment is required to identify the underlying cause.

In the Comella systematic review of oesophageal morbidity, it was noted that a younger age was associated with higher rates of swallow dysfunction [4]. Two studies included in our review reported results by age [16, 17]. Maybee and colleagues identified decreasing rates of difficulty swallowing liquids but increasing rates of difficulty swallowing solids foods with increasing age [17]. Comparison of pooled prevalence rates for patient/parent-reported difficulty swallowing solid foods in our review identified slightly higher prevalence in studies only including adult participants (50%) compared to those including only child participants (43%). Differences may be due to reporting method (i.e. instrumental vs non-instrumental assessment), or study population. It is evidence that questions remain regarding potential improvement in function with age.

### Psychosocial impact

Evidence from both quantitative and qualitative data indicates that eating, drinking and swallowing difficulties in OA/TOF can have psychosocial, as well as health impacts. This phenomenon has been more widely explored with parents/carers than for individuals born with OA/TOF. The development of a condition-specific QOL tool for children, which includes consideration of eating and drinking, has resulted in exploration of eating and drinking-related QOL [77]. However, relatively little is reported in the literature as to whether or how OA/TOF related swallow dysfunction impacts on eating and drinking-related QOL in adults. Evidence from the qualitative study included in this review suggests that adults born with OA/TOF experience anxiety related specifically to eating situations, which impact on their ability to enjoy meals out or social eating situations [70]. Several tools exist that specifically investigate these important aspects of QOL, such as the SWAL-QOL [78] and MDADI [79], which could be adopted in clinical practice to ensure this important aspect of care is addressed, as well as a new condition-specific QOL tool [80].

For children it is often parent QOL that is impacted by feeding difficulties [81]. Evidence from both quantitative and qualitative studies suggests that eating and drinking-related QOL is significantly affected for parents of children with OA/TOF. For young children, eating and drinking is a dyadic process, with the parent integral to the child’s experience. Therefore, it is paramount that we consider the needs of parents in routine clinical practice, alongside those of the child, to optimise eating and drinking outcomes.

### Limitations

Numerous conditions associated with swallow dysfunction and eating/drinking difficulties are known to co-occur with OA/TOF, such as cardiac abnormalities, structural airway abnormalities, gastro-oesophageal reflux and prematurity. Likewise, numerous factors associated with OA subtype or repair type have the potential to impact on outcome. Subgroup analysis to assess the impact of OA subtype, repair type, co-morbidities and the impact of late introduction to oral feeding on eating, drinking and swallowing outcomes was not possible due to varied reporting. As has been suggested previously, national or international registries with prospectively collected data for a wide range of outcomes would be required to answer these questions [4].

## Conclusions

This review suggests that prevalence of oral and pharyngeal phase swallowing difficulties may be as high as 24% in children with repaired OA/TOF. In this population, swallowing difficulties present as altered eating and drinking behaviours, most commonly: the need to drink water when eating (49%), prolonged mealtimes (37%) and the need to modify food or drink textures (28%) across the lifespan. These swallowing difficulties can impact on psychological well-being and quality of life, both for the individual and for parents/other family members. The complex interaction of multi-phase changes in swallow physiology and subsequent impact on well-being warrants specialist, multi-disciplinary, long-term follow-up to optimise outcomes.

### Key practice and research recommendations

- Use of quantitative or valid measures of oro-pharyngeal swallow dysfunction using instrumental assessment.
- Consider inclusion of routine assessment of oro-pharyngeal swallow dysfunction for all individuals with OA/TOF across the life-span but particularly under 1’s.
- Ensure specialist, multi-disciplinary assessment and management of feeding and swallowing difficulties across the lifespan.
- Ensure accurate description of “dysphagia” reporting in outcome studies, with differentiation between oro-pharyngeal and oesophageal swallow dysfunction.
- Agree definition and terminology for “feeding difficulty” to determine true nature and prevalence. Consider adopting definition and diagnostic criteria of “pediatric feeding disorder” [82].
- Ensure eating and drinking difficulties are viewed holistically, ensuring optimised swallow function and eating/drinking quality of life for the individual and family.

### Supplementary Information

Supplementary Material 1.

Supplementary Material 2.

## Data Availability

Reference and data extraction files containing the raw data from which analysis was undertaken can be found here: 10.5522/04/25920946.v1.
