# Supplementary material for: The characteristics of eating, drinking and oro-pharyngeal swallowing difficulties associated with repaired oesophageal atresia/tracheo-oesophageal fistula: a systematic review and meta-proportional analysis
Source: Orphanet J Rare Dis. 2024 Jul 4;19:253. doi: 10.1186/s13023-024-03259-x (PMC11225380; doi:10.1186/s13023-024-03259-x)
Supplement: Supplementary file 1 — Supplementary Material 1. [file 13023_2024_3259_MOESM1_ESM.docx]

Supplementary material 1

Data extraction

| Authors |
| --- |
| Date |
| Country |
| Title |
| Study design |
| Participant demographics: age at assessment, type of oa/tof, age at repair, type of repair, co-morbidities |
| Inclusion/exclusion criteria |
| Participant recruitment |
| Sample size |
| Assessment type (e.g., videofluoroscopy, parent reported questionnaire, notes review) |
| Outcome measures used |
| Description of assessment results (e.g. Videofluoroscopy findings, questionnaire results, qualitative themes) |
| Prevalence of dysphagia or eating/drinking/mealtime characteristics |
| From qualitative studies “findings” were extracted: researcher themes or interpretations accompanied by illustrative quotes |
