# Supplementary material for: The characteristics of eating, drinking and oro-pharyngeal swallowing difficulties associated with repaired oesophageal atresia/tracheo-oesophageal fistula: a systematic review and meta-proportional analysis
Source: Orphanet J Rare Dis. 2024 Jul 4;19:253. doi: 10.1186/s13023-024-03259-x (PMC11225380; doi:10.1186/s13023-024-03259-x)
Supplement: Supplementary file 2 — Supplementary Material 2. [file 13023_2024_3259_MOESM2_ESM.docx]

| **Author and year** | **Country** | **Study type** | **Participant age** | **Repair type** | **Number of participants** | **Gross Type N** | | | | | **Type of assessment** | **MMAT** |
| --- | --- | --- | --- | --- | --- | --- | --- | --- | --- | --- | --- | --- |
|  |  |  |  |  |  | **A** | **B** | **C** | **D** | **E** |  |  |
| Maybee 2023 | USA | Case series | <1 year; 1-4 years; 5-11 years; 11-18 years | NR | 44 | NR | NR | NR | NR | NR | Instrumental Non-instrumental | 57% |
| Gibreel 2017 | USA | Cross sectional | >18 years | Immediate primary; Oesophageal replacement; Delayed primary | 46 | 4 | 1 | 40 | 0 | 1 | Non-instrumental | 100% |
| Demir 2017 | Turkey | Case control | 1-4 years | Immediate primary; Delayed primary | 18 | 12 | 1 | 5 | 0 | 0 | Instrumental | 29% |
| Harrington 2021 | USA | Case series | 1-4 years | Delayed primary | 45 | 39 | 12 | 7 | 0 | 0 | Non-instrumental | 86% |
| Thompson 2021 | USA | Case series | 1-4 years; 5-11 years | Oesophageal replacement | 41 | 11 | 4 | 20 | 0 | 0 | Non-instrumental | 86% |
| DiNatale 2022 | Switzerland | Cross sectional | 5-11 years; 11-18 years; >18 years | Immediate primary; Oesophageal replacement; Delayed primary | 30 | 5 | 0 | 25 | 0 | 0 | Non-instrumental | 43% |
| Bergmann 2022 | Germany | Cross sectional | 1-4 years; 5-11 years; 11-18 years | Immediate primary; Delayed primary | 44 | 6 | 1 | 36 | NR | NR | Non-instrumental | 43% |
| Puntis 1990 | UK | Cross sectional | <1 year; 1-4 years; 5-11 years; 11-18 years; >18 years | Immediate primary; Oesophageal replacement | 124 | NR | NR | NR | NR | NR | Non-instrumental | 55% |
| Cavallaro 1992 | Italy | Cross sectional | 1-4 years; 5-11 years | Immediate primary; Delayed primary | 28 | 4 | 0 | 23 | 0 | 1 | Non-instrumental | 57% |
| Chetcuti 1993 | Australia | Cross sectional | <1 year; 1-4 years; 5-11 years; 11-18 years; >18 years | Immediate primary; Oesophageal replacement; Delayed primary | 334 | 16 | 6 | 290 | 0 | 22 | Non-instrumental | 14% |
| Ure 1995 | Germany | Case series | >18 years | Oesophageal replacement | 8 | 3 | 1 | 5 | 0 | 0 | Non-instrumental | 71% |
| Montgomery 1998 | Sweden | Cross sectional | >18 years | NR | 11 | NR | NR | NR | NR | NR | Instrumental | 57% |
| Krug 1999 | Netherlands | Cross sectional | >18 years | Immediate primary; Delayed primary | 39 | NR | NR | NR | NR | NR | Non-instrumental | 43% |
| Schier 2001 | Germany | Cross sectional | 5-11 years; 11-18 years; >18 years | Immediate primary; Oesophageal replacement; Delayed primary | 128 | 14 | 9 | 93 | 6 | 0 | Non-instrumental | 57% |
| Hormann 2002 | Austria | Case series | <1 year; 1-4 years; 5-11 years | NR | 19 | 0 | 0 | 18 | 1 | 0 | Instrumental | 43% |
| Deurloo 2003 | Netherlands | Cross sectional | >18 years | Immediate primary; Oesophageal replacement | 38 | 1 | 0 | 39 | 0 | 0 | Non-instrumental | 86% |
| Deurloo 2005 | Netherlands | Cross sectional | 11-18 years; >18 years | NR | 86 | NR | NR | NR | NR | NR | Non-instrumental | 71% |
| Cimador 2006 | Italy | Case series | 11-18 years | Immediate primary | 15 | 0 | 0 | 15 | 0 | 0 | Non-instrumental | 43% |
| Taylor 2007 | Australia | Cross sectional | >18 years | NR | 132 | NR | NR | NR | NR | NR | Non-instrumental | 57% |
| Golonka 2008 | Canada | Case series | <1 year | Delayed primary | 4 | 2 | 1 | 1 | 0 | 0 | Instrumental | 29% |
| Frohlich 2008 | Germany | Cross sectional | <1 year; 1-4 years; 5-11 years; 11-18 years; >18 years | NR | 24 | 1 | 0 | 21 | 1 | 1 | Non-instrumental | 43% |
| Faugli 2008 | Norway | Case control study | <1 year; 1-4 years | NR | 37 | NR | NR | NR | NR | NR | Non-instrumental | 86% |
| Sistonen 2010 | Finland | Cross sectional | >18 years | Immediate primary; Delayed primary | 101 | 0 | 2 | 91 | 5 | 3 | Non-instrumental | 86% |
| Castilloux 2010 | Canada | Cross sectional | 1-4 years; 5-11 years | Immediate primary; Delayed primary | 45 | NR | NR | 37 | NR | NR | Non-instrumental | 57% |
| Gatzinsky 2011 | Sweden | Cross sectional | >18 years | Immediate primary; Oesophageal replacement; Delayed primary | 73 | 3 | 1 | 69 | 1 | 5 | Non-instrumental | 86% |
| Legrand 2012 | France | Cross sectional | 5-11 years; 11-18 years | Immediate primary; Delayed primary | 57 | 0 | 0 | 57 | 0 | 0 | Non-instrumental | 71% |
| Lemoine 2013 | Canada | Case series | <1 year; 1-4 years; 5-11 years; 11-18 years | NR | 40 | 5 | 0 | 35 | 0 | 0 | Non-instrumental | 43% |
| Baird 2015 | Canada | Case series | <1 year; 1-4 years; 5-11 years | Immediate primary; Oesophageal replacement; Delayed primary | 30 | 2 | 0 | 26 | 1 | 1 | Non-instrumental | 57% |
| Fraga 2015 | USA | Case series | <1 year; 1-4 years; 5-11 years; 11-18 years | Immediate primary | 22 | 0 | 0 | 19 | 0 | 3 | Instrumental | 71% |
| HuynhTrudeau 2015 | Canada | Cross sectional | >18 years | Immediate primary; Oesophageal replacement | 41 | NR | NR | 35 | NR | NR | Non-instrumental | 71% |
| Tan 2015 | China | Case series | 1-4 years; 5-11 years | NR | 7 | NR | NR | NR | NR | NR | Non-instrumental | 71% |
| Yalcin 2015 | Turkey | Case series | <1 year; 1-4 years; 5-11 years | Immediate primary; Delayed primary | 32 | 3 | 0 | 26 | 2 | 1 | Instrumental | 43% |
| Presse 2016 | Canada | Case series | >18 years | Immediate primary; Oesophageal replacement | 37 | 6 | 0 | 31 | 0 | 0 | Non-instrumental | 71% |
| Coppens 2016 | Netherlands | Case series | <1 year; 1-4 years; 5-11 years; 11-18 years | NR | 111 | 9 | 1 | 86 | 6 | 6 | Instrumental | 71% |
| Barni 2019 | Italy | Case report | <1 year | Immediate primary | 1 | 0 | 0 | 1 | 0 | 0 | Non-instrumental | 50% |
| Menzies 2017 | Australia | Case series | <1 year; 1-4 years; 5-11 years; 11-18 years | Immediate primary; Delayed primary | 56 | 5 | 10 | 53 | 1 | 1 | Non-instrumental | 57% |
| Dellenmark-Blom 2019 | Sweden | Cross sectional | 1-4 years; 5-11 years; 11-18 years | Immediate primary; Oesophageal replacement; Delayed primary | 116 | NR | NR | NR | NR | NR | Non-instrumental | 57% |
| Arslan 2020 | Turkey | Non-randomised experimental study | 1-4 years | Immediate primary; Oesophageal replacement; Delayed primary | 20 | 9 | 0 | 11 | 0 | 0 | Non-instrumental | 43% |
| Dellenmark-Blom 2020 | Sweden, Germany | Cross sectional | 1-4 years; 5-11 years; 11-18 years | Immediate primary; Oesophageal replacement; Delayed primary | 124 | NR | NR | NR | NR | NR | Non-instrumental | 71% |
| Serel Arslan 2020 | Turkey | Cross sectional | 1-4 years; 5-11 years; 11-18 years | Immediate primary; Oesophageal replacement; Delayed primary | 64 | 28 | 0 | 36 | 0 | 0 | Non-instrumental | 57% |
| Bevilacqua 2020 | Italy | Cross sectional | 1-4 years | NR | 51 | 6 | 2 | 43 | 0 | 0 | Non-instrumental | 43% |
| Birketvedt 2020 | Norway | Cross sectional | 11-18 years; >18 years | NR | 68 | 3 | 0 | 58 | 4 | 3 | Non-instrumental | 71% |
| Menzies 2020 | Australia | Cross sectional | 1-4 years; 5-11 years | NR | 20 | NR | NR | 1 9 | NR | NR | Non-instrumental | 57% |
| Ax 2021 | Sweden | Cross sectional | 1-4 years; 5-11 years; 11-18 years | NR | 114 | 9 | 5 | 93 | 2 | 5 | Non-instrumental | 43% |
| Rabone 2021 | UK | Cross sectional | >18 years | NR | 92 | NR | NR | 54 | NR | NR | Non-instrumental | 100% |
| vanTuyllvan  Serooskerken 2021 | Netherlands | Case series | 1-4 years; 5-11 years; 11-18 years | Delayed primary | 11 | 6 | 5 | 0 | 0 | 0 | Non-instrumental | 86% |
| Pham 2022 | France | Cross sectional | 1-4 years | NR | 145 | 7 | NR | 133 | NR | NR | Non-instrumental | 100% |
| Dellenmark-Blom 2022 | Sweden, Germany | Cross sectional | 1-4 years; 5-11 years; 11-18 years | Immediate primary ; Oesophageal replacement; Delayed primary | 180 | 24 | 0 | NR | NR | NR | Non-instrumental | 71% |
| Dellenmark-Blom 2022 | Sweden | Cross sectional | 1-4 years; 5-11 years; 11-18 years | Delayed primary; Oesophageal replacement | 30 | 12 | 8 | 10 | 0 | 0 | Non-instrumental | 86% |
| Traini 2022 | Australia | Cross sectional | 1-4 years; 5-11 years; 11-18 years | Immediate primary ; Delayed primary | 21 | NR | NR | 14 | NR | NR | Non-instrumental | 71% |
| Celtik 2022 | Turkey | Cross sectional | <1 year; 1-4 years; 5-11 years; 11-18 years | Immediate primary ; Oesophageal replacement | 27 | 6 | 3 | 17 | 0 | 0 | Instrumental | 43% |
| Stewart 2022 | UK | Qualitative research | <1 year; 1-4 years; 5-11 years; 11-18 years | Immediate primary; Delayed primary; Oesophageal replacement | 127 | 3 | NR | 77 | NR | 4 | Non-instrumental | 100% |
| Wallace 2022 | UK | Qualitative research | <1 year; 1-4 years; 5-11 years | NR | 176 | NR | NR | NR | NR | NR | Non-instrumental | 100% |
| Capitanio 2021 | Italy | Cross sectional | 5-11 years; 11-18 years | NR | 50 | 5 | 2 | 42 | 1 | 0 | Non-instrumental | 29% |
| Soyer 2022 | Turkey | Case series | <1 year; 1-4 years | Immediate primary; Oesophageal replacement; Delayed primary | 55 | 18 | 1 | 29 | 1 | 0 | Instrumental | 43% |
| Bourg 2022 | France | Cohort study | 5-11 years | Immediate primary; Oesophageal replacement; Delayed primary | 93 | NR | NR | NR | NR | NR | Non-instrumental | 43% |
| Yasuda 2022 | USA | Cross sectional | <1 year; 1-4 years; 5-11 years | Immediate primary; Delayed primary | 330 | NR | NR | NR | NR | NR | Non-instrumental | 57% |
| Mikkelsen 2022 | Norway | Cross sectional | 11-18 years; >18 years | Immediate primary; Oesophageal replacement | 68 | 3 | 0 | 58 | 4 | 3 | Non-instrumental | 100% |
| Leibovitch 2018 | Isreal | Cross sectional | 1-4 years; 5-11 years; 11-18 years; >18 years | NR | 46 | NR | NR | NR | NR | NR | Non-instrumental | 43% |
| Baxter 2018 | USA | Case series | 1-4 years | NR | 145 | 6 | 2 | 125 | 1 | 11 | Non-instrumental | 86% |
| Fung 2019 | Canada | Case series | NR | NR | 197 | 13 | 5 | 162 | 1 | 16 | Instrumental | 71% |
| Serel Arslan 2017 | Turkey | Randomised controlled trial | <1 year; 1-4 years | Immediate primary; Delayed primary | 24 | 8 | 0 | 16 | 0 | 0 | Instrumental | 57% |
| Soyer 2017 | Turkey | Case series | 1-4 years; 5-11 years | Immediate primary; Delayed primary | 40 | 5 | 0 | 36 | 0 | 0 | Instrumental | 43% |
| Serel Arslan 2018 | Turkey | Cross sectional | 1-4 years; 5-11 years; 11-18 years | Immediate primary; Oesophageal replacement; Delayed primary | 30 | 12 | 0 | 18 | 0 | 0 | Non-instrumental | 43% |
| Svoboda 2018 | International | Cross sectional | <1 year; 1-4 years; 5-11 years; 11-18 years; >18 years | NR | 928 | 176 |  | 742 |  | 9 | Non-instrumental | 57% |
